# 

**Published:** 1862-05

**Authors:** 


### BOOKS AND MAGAZINES RECEIVED.

*On Bandaging and other Operations of Minor Surgery; by* F. W. SARGENT, M. D., *member of the College of Physicians of Philadelphia, one of the Consulting Surgeons to Wells' Hospital—new edition with an additional chapter on Military Surgery by* W. F. ATLEE, M. D., *and one hundred and eighty-seven illustrations. Philadelphia:* BLANCHARD & LEA, 1862.

*Peterson's Magazine. Published by* CHARLES J. PETERSON, 306 *Chesnut Street, Philadelphia.*

This Magazine is highly embellished with beautiful plates, and always contains very readable matter, interesting to those for whom it is intended, viz: the ladies, the wives, the daughters, and the children, and even the fathers might profitably beguile a leisure hour in its perusal, though we *do not* propose to recommend it to the careful study of the medical profession.

*Harpers' New Monthly Magazine for May. Published by* HARPER & BROTHERS, *Franklin Square, New York.*

This Magazine comes regularly upon our table, and contains an interesting variety of very readable and instructive articles. An editor's wife and daughters (if he has any) are more interested in exchanges of this description than almost any other. The May number we have read ourselves, and find it particularly interesting. What has most attracted us, is the chapter upon the life and character of Dr. Valentine Mott, giving a sketch of his early life and history, and many events in his travels in England, France and Germany.

---

### PAMPHLETS RECEIVED.

*Valedictory Address to the Graduating Class of the Cincinnati College of Medicine and Surgery, at the close of Session* 1861–2. *Delivered February* 12. *By* A. H. BAKER, M. D., *Professor of Surgery.*

*Ten Lectures on Medical Electricity, embracing the System of Electropathic Treatment, by* P. J. BECKER. *Auburn,* 1862.

*A Description of the newly-invented Elastic Tourniquet, for the use of Armies and employment of civil life; its uses and applications, with Remarks on the Different Methods of Arresting Hæmorrhage from Gunshot and other Wounds. New York:* GEORGE F. NESBITT & Co., 1862.

AMERICAN MEDICAL ASSOCIATION—ANNUAL MEETING.—We, the undersigned, Committee of Arrangements of the American Medical Association, after free consultation with officers and members in each important section of the country, accessible to the Committee, feel constrained to give notice to the profession, that the regular Annual Meeting of the Association is further *postponed* until the first Tuesday in June, 1863.

*Chicago, April 5th*, 1862.

N. S. DAVIS, J. BLOODGOOD,
J. W. FREER, D. LASKIE MILLER,
H. H. JONES, E. ANDREWS,
THOS. BEVAN, *Committee.*

---

The Metropolitan Health Bill has passed one branch of the New York Legislature, and has met with unexpected difficulties in the way of proposed amendments. It will be quite impossible to give such a measure the political shade which mere politicians desire, and we greatly fear that any changes at this late day will prove fatal to its enactment.—*Med. Times.*

